# Loss-of-function nuclear factor κB subunit 1 *(NFKB1)* variants are the most common monogenic cause of common variable immunodeficiency in Europeans

**DOI:** 10.1016/j.jaci.2018.01.039

**Published:** 2018-10

**Authors:** Paul Tuijnenburg, Hana Lango Allen, Siobhan O. Burns, Daniel Greene, Machiel H. Jansen, Emily Staples, Jonathan Stephens, Keren J. Carss, Daniele Biasci, Helen Baxendale, Moira Thomas, Anita Chandra, Sorena Kiani-Alikhan, Hilary J. Longhurst, Suranjith L. Seneviratne, Eric Oksenhendler, Ilenia Simeoni, Godelieve J. de Bree, Anton T.J. Tool, Ester M.M. van Leeuwen, Eduard H.T.M. Ebberink, Alexander B. Meijer, Salih Tuna, Deborah Whitehorn, Matthew Brown, Ernest Turro, Adrian J. Thrasher, Kenneth G.C. Smith, James E. Thaventhiran, Taco W. Kuijpers, NIHR BioResource–Rare Diseases Consortium, Zoe Adhya, Hana Alachkar, Ariharan Anantharachagan, Richard Antrobus, Gururaj Arumugakani, Chiara Bacchelli, Helen Baxendale, Claire Bethune, Shahnaz Bibi, Barbara Boardman, Claire Booth, Michael Browning, Mary Brownlie, Siobhan Burns, Anita Chandra, Hayley Clifford, Nichola Cooper, Sophie Davies, John Dempster, Lisa Devlin, Rainer Doffinger, Elizabeth Drewe, David Edgar, William Egner, Tariq El-Shanawany, Bobby Gaspar, Rohit Ghurye, Kimberley Gilmour, Sarah Goddard, Pavel Gordins, Sofia Grigoriadou, Scott Hackett, Rosie Hague, Lorraine Harper, Grant Hayman, Archana Herwadkar, Stephen Hughes, Aarnoud Huissoon, Stephen Jolles, Julie Jones, Peter Kelleher, Nigel Klein, Taco Kuijpers, Dinakantha Kumararatne, James Laffan, Hana Lango Allen, Sara Lear, Hilary Longhurst, Lorena Lorenzo, Jesmeen Maimaris, Ania Manson, Elizabeth McDermott, Hazel Millar, Anoop Mistry, Valerie Morrisson, Sai Murng, Iman Nasir, Sergey Nejentsev, Sadia Noorani, Eric Oksenhendler, Mark Ponsford, Waseem Qasim, Ellen Quinn, Isabella Quinti, Alex Richter, Crina Samarghitean, Ravishankar Sargur, Sinisa Savic, Suranjith Seneviratne, Carrock Sewall, Fiona Shackley, Ilenia Simeoni, Kenneth G.C. Smith, Emily Staples, Hans Stauss, Cathal Steele, James Thaventhiran, Moira Thomas, Adrian Thrasher, Steve Welch, Lisa Willcocks, Sarita Workman, Austen Worth, Nigel Yeatman, Patrick Yong, Sofie Ashford, John Bradley, Debra Fletcher, Tracey Hammerton, Roger James, Nathalie Kingston, Willem Ouwehand, Christopher Penkett, F Lucy Raymond, Kathleen Stirrups, Marijke Veltman, Tim Young, Sofie Ashford, Matthew Brown, Naomi Clements-Brod, John Davis, Eleanor Dewhurst, Marie Erwood, Amy Frary, Rachel Linger, Jennifer Martin, Sofia Papadia, Karola Rehnstrom, William Astle, Antony Attwood, Marta Bleda, Keren Carss, Louise Daugherty, Sri Deevi, Stefan Graf, Daniel Greene, Csaba Halmagyi, Matthias Haimel, Fengyuan Hu, Roger James, Hana Lango Allen, Vera Matser, Stuart Meacham, Karyn Megy, Christopher Penkett, Olga Shamardina, Kathleen Stirrups, Catherine Titterton, Salih Tuna, Ernest Turro, Ping Yu, Julie von Ziegenweldt, Abigail Furnell, Rutendo Mapeta, Ilenia Simeoni, Simon Staines, Jonathan Stephens, Kathleen Stirrups, Deborah Whitehorn, Paula Rayner-Matthews, Christopher Watt

**Affiliations:** a Department of Pediatric Hematology, Immunology and Infectious Diseases, Emma Children's Hospital, Academic Medical Center, Amsterdam, The Netherlands; b Department of Experimental Immunology, Academic Medical Center, Amsterdam, The Netherlands; k Department of Internal Medicine, Academic Medical Center, Amsterdam, The Netherlands; c Department of Haematology, University of Cambridge, Cambridge, United Kingdom; f Department of Medicine, University of Cambridge, Cambridge, United Kingdom; d NHS Blood and Transplant Cambridge, Cambridge Biomedical Campus, Cambridge, United Kingdom; o NIHR BioResource–Rare Diseases, Cambridge Biomedical Campus, Cambridge, United Kingdom; e Department of Immunology, Royal Free London NHS Foundation Trust, University College London Institute of Immunity and Transplantation, London, United Kingdom; g Department of Immunology, Queen Elizabeth University Hospital, Glasgow, United Kingdom; h Department of Immunology, Royal Surrey County Hospital, Guildford, United Kingdom; i Department of Immunology, Barts Health NHS Trust, London, United Kingdom; j Department of Clinical Immunology, Hôpital Saint-Louis, Assistance Publique Hôpitaux de Paris (APHP), Paris, France; l Department of Blood Cell Research, Sanquin Research, Amsterdam, The Netherlands; m Department of Plasma Proteins, Sanquin Research, Amsterdam, The Netherlands; n Molecular and Cellular Immunology Section, UCL Great Ormond Street Institute of Child Health and Great Ormond Street Hospital NHS Trust London, London, United Kingdom

**Keywords:** B cells, common variable immunodeficiency, nuclear factor κB1, CFSE, Carboxyfluorescein succinimidyl ester, CVID, Common variable immunodeficiency, LOF, Loss-of-function, NF-κB, Nuclear factor κB, NFKB1, Nuclear factor κB subunit 1, NIHRBR-RD, NIHR BioResource–Rare Diseases, PID, Primary immunodeficiency disease, PML, Progressive multifocal leukoencephalopathy, RHD, Rel homology domain, VEP, Variant effect predictor

## Abstract

**Background:**

The genetic cause of primary immunodeficiency disease (PID) carries prognostic information.

**Objective:**

We conducted a whole-genome sequencing study assessing a large proportion of the NIHR BioResource–Rare Diseases cohort.

**Methods:**

In the predominantly European study population of principally sporadic unrelated PID cases (n = 846), a novel Bayesian method identified nuclear factor κB subunit 1 *(NFKB1)* as one of the genes most strongly associated with PID, and the association was explained by 16 novel heterozygous truncating, missense, and gene deletion variants. This accounted for 4% of common variable immunodeficiency (CVID) cases (n = 390) in the cohort. Amino acid substitutions predicted to be pathogenic were assessed by means of analysis of structural protein data. Immunophenotyping, immunoblotting, and *ex vivo* stimulation of lymphocytes determined the functional effects of these variants. Detailed clinical and pedigree information was collected for genotype-phenotype cosegregation analyses.

**Results:**

Both sporadic and familial cases demonstrated evidence of the noninfective complications of CVID, including massive lymphadenopathy (24%), unexplained splenomegaly (48%), and autoimmune disease (48%), features prior studies correlated with worse clinical prognosis. Although partial penetrance of clinical symptoms was noted in certain pedigrees, all carriers have a deficiency in B-lymphocyte differentiation. Detailed assessment of B-lymphocyte numbers, phenotype, and function identifies the presence of an increased CD21^low^ B-cell population. Combined with identification of the disease-causing variant, this distinguishes between healthy subjects, asymptomatic carriers, and clinically affected cases.

**Conclusion:**

We show that heterozygous loss-of-function variants in *NFKB1* are the most common known monogenic cause of CVID, which results in a temporally progressive defect in the formation of immunoglobulin-producing B cells.

## Graphical abstract

**Figure:**
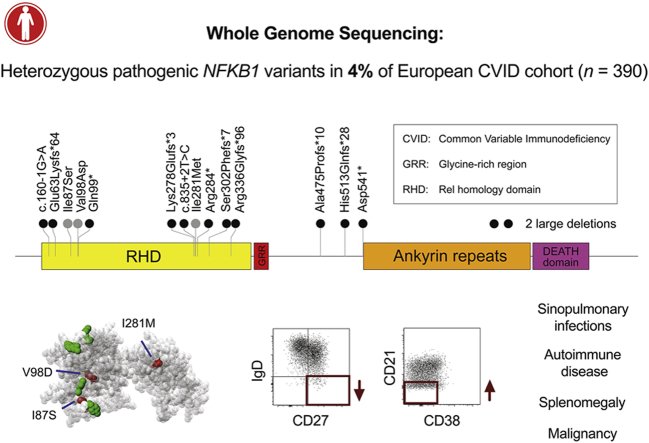

Common variable immunodeficiency (CVID; MIM 607594), which occurs in approximately 1:25,000 persons^1^, ^2^, ^3^ is a clinically and genetically heterogeneous disorder characterized by susceptibility to sinopulmonary infections, hypogammaglobulinemia, and poor vaccine responses. CVID is the most common primary immune deficiency, requiring lifelong clinical follow-up, and the clinical course is highly variable, with substantial excess mortality. Affected subjects present frequently with recurrent respiratory tract infections, as well as immune dysregulatory features. The antibody deficiency is often not as marked as the agammaglobulinemia seen in patients with genetically defined conditions leading to B-lymphocyte aplasia.^4^, ^5^ Conversely, although patients with B-lymphocyte aplasia have a favorable prognosis on adequate replacement immunoglobulin treatment, the response of patients with CVID is highly variable.

Past studies focused on familial cases with CVID and used techniques ranging from traditional linkage analysis to more recent exome sequencing to characterize the genetic cause. This has revealed that monogenic gene dysfunction accounts for 10% of cases.^4^, ^5^ Several of the variants in these genes have been characterized as partially penetrant; it remains unclear whether genetic or environmental factors determine disease onset. Multiple recent studies identified variants in nuclear factor κB subunit 1 *(NFKB1)* as a monogenic cause of CVID and reported on the clinical features of these cases.^6^, ^7^, ^8^, ^9^, ^10^, ^11^

As part of this NIHR BioResource–Rare Diseases study, we sequenced the genomes of 846 unrelated patients with predominantly sporadic primary immunodeficiency disease (PID) who were recruited from across the United Kingdom and by European collaborators. Application of the recently developed statistical method BeviMed^12^ to the 846 PID cases and more than 5000 control genomes identified *NFKB1* as the gene with the highest probability of association with the disease and with the largest number of cases explained by variants in that gene. Further investigations revealed a series of 16 heterozygous loss-of-function (LOF) variants in *NFKB1* as the most common genetic cause of CVID.

Mutations in genes that affect nuclear factor κB (NF-κB)–dependent signaling are associated with a number of immunodeficiencies.^13^, ^14^, ^15^, ^16^, ^17^, ^18^, ^19^, ^20^, ^21^, ^22^, ^23^, ^24^, ^25^, ^26^ NF-κB is a ubiquitous transcription factor member of the Rel proto-oncogene family. NF-κB regulates the expression of several genes involved in inflammatory and immune responses. The classical activated form of NF-κB consists of a heterodimer of the p50/p65 protein subunits. The NF-κB family of transcription factors comprises 5 related proteins, c-Rel, p65 (RelA), RelB, p50 (NF-κB1) and p52 (NF-κB2), which interact to form homodimers and heterodimers with distinct gene regulatory functions.^13^, ^27^, ^28^, ^29^ Each Rel NF-κB protein has a conserved 300-amino-acid N-terminal Rel homology domain (RHD) that encompasses sequences needed by NF-κB proteins to bind DNA motifs (κB elements), form dimers, interact with regulatory inhibitor IκB proteins, and enter the nucleus. The 10 different NF-κB dimers identified have distinct transcriptional properties.^28^ In most cells NF-κB is retained in the cytosol in a latent state through interaction with the IκB proteins (eg, IκBα, IκBβ, and IκBε), a family of proteins with ankyrin repeats that mediate IκB interaction with the RHD of NF-κB, masking the nuclear localization sequence and DNA-binding domains. Signal-dependent activation of an IκB kinase complex comprising catalytic (α and β) and regulatory (NF-κB essential modulator) subunits induces the phosphorylation and degradation of IκB,^29^ which permits NF-κB factors to enter the nucleus and regulate gene expression.

We show that variants in *NFKB1* culminate in a progressive humoral immunodeficiency indistinguishable from CVID, with a highly variable penetrance. We demonstrate the utility of an *in silico* protein prediction model for validating the predicted disease-causing substitutions, and we report on the clinical spectrum, immunologic phenotype, and functional consequences of heterozygous *NFKB1* variants.

## Methods

### Cohort

The NIHR BioResource–Rare Diseases (NIHRBR-RD) study was established in the United Kingdom to further the clinical management of patients with rare diseases by providing a national resource of whole-genome sequence data. All participants provided written informed consent, and the study was approved by the East of England–Cambridge South national institutional review board (13/EE/0325). At the time of our analysis, the NIHRBR-RD study included whole-genome sequence data from 8066 subjects, of whom 1299 were part of the PID cohort. These were predominantly singleton cases, but additional affected and/or unaffected family members of some of the patients were also sequenced; in total, there were 846 unrelated index cases.

Patients with PIDs were recruited by specialists in clinical immunology (either trained in pediatrics or internal medicine) from 26 hospitals in the United Kingdom and a smaller number came from The Netherlands, France, and Italy. The recruitment criteria included the following: clinical diagnosis of CVID according to the European Society for Immunodeficiencies ESID criteria (ESID Registry–Working Definitions for Clinical Diagnosis of PID, 2014, latest version: April 25, 2017), extreme autoimmunity, or recurrent (and/or unusual) infections suggestive of severely defective innate or cell-mediated immunity. Exclusion of known causes of PID was encouraged, and some of the patients were screened for 1 or more PID genes before enrollment in the PID cohort. The ethnic makeup of the study cohort represented that of the general United Kingdom population: 82% were European, 6% were Asian, 2% were African, and 10% were of mixed ethnicity based on the patients’ whole-genome data.

Given that PID is a heterogeneous disease, with overlap in phenotypes and genetic causes across different diagnostic categories, we decided to perform an unbiased genetic analysis of all 846 unrelated index cases. Whole-genome sequence data were additionally available for 63 affected and 345 unaffected relatives. Within a broad range of phenotypes, CVID is the most common disease category, comprising 46% of the NIHRBR-RD PID cohort (n = 390 index cases; range, 0-93 years of age).

### Sequencing and variant filtering

Whole-genome sequencing of paired-end reads was performed by Illumina on their HiSeq X Ten system (Illumina, San Diego, Calif). Reads of 100, 125, or 150 bp in length were aligned to the GRCh37 genome build by using the Isaac aligner, variants across the samples were jointly called with the AGG tool, and large deletions were identified by using Canvas and Manta algorithms (all software by Illumina), as described previously.^30^ Average read depth was 35, with 95% of the genome covered by at least 20 reads.

Single nucleotide variants and small insertions/deletions were filtered based on the following criteria: passing standard Illumina quality filters in greater than 80% of the genomes sequenced by the NIHRBR-RD study, having a variant effect predictor (VEP)^31^ effect of either moderate or high, having a minor allele frequency less than 0.001 in the Exome Aggregation Consortium data set, and having a minor allele frequency of less than 0.01 in the NIHRBR-RD cohort. Large deletions called by both Canvas and Manta algorithms, passing standard Illumina quality filters, overlapping at least 1 exon absent from control data sets,^32^ and having a frequency of less than 0.01 in the NIHRBR-RD genomes were included in the analysis.

**Fig E1:**
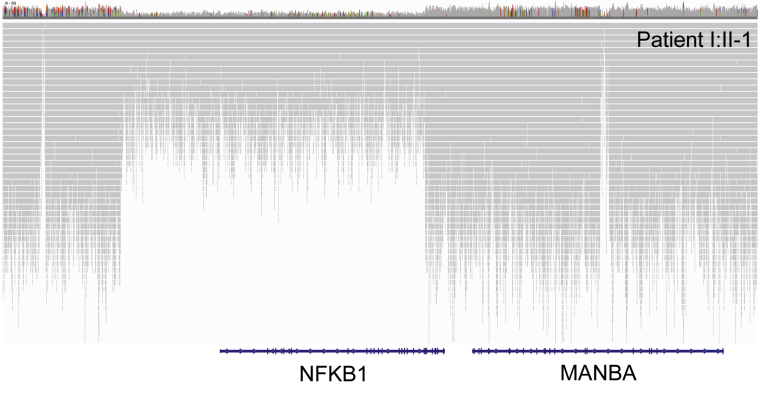


All variants reported as disease causing in this study were confirmed by using Sanger sequencing with standard protocols. Large deletions were inspected in the Integrative Genomics Viewer plot (see Fig E1 in this article's Online Repository at www.jacionline.org), and breakpoints were confirmed by sequencing the PCR products spanning each deletion.

### Gene and variant pathogenicity estimation

To evaluate genes for their association with PID, we applied the BeviMed inference procedure^12^ to the NIHRBR-RD whole-genome data set. BeviMed (https://CRAN.R-project.org/package=BeviMed) evaluates the evidence for association between case/control status of unrelated subjects and allele counts at rare variant sites in a given locus. The method infers the posterior probabilities of association under dominant and recessive inheritance and, conditional on such an association, the posterior probability of pathogenicity of each considered variant in the locus. BeviMed was applied to rare variants and large rare deletions in each gene, treating the 846 unrelated PID index cases as cases and the 5097 unrelated subjects from the rest of the NIHRBR-RD cohort as control subjects. All genes were assigned the same prior probability of association with the disease of .01, regardless of their previously published associations with an immune deficiency phenotype. Variants with a VEP effect labeled as high were assigned higher prior probabilities of pathogenicity than variants with a moderate effect, as described previously.^12^

### Immunophenotyping and B- and T-cell functional assays

PBMCs were isolated by using standard density gradient centrifugation techniques with Lymphoprep (Nycomed, Oslo, Norway). Absolute numbers of lymphocytes, T cells, B cells, and natural killer cells were determined with Multitest 6-color reagents (BD Biosciences, San Jose, Calif), according to the manufacturer's instructions. For PBMC immunophenotyping, we refer to the Methods section in this article's Online Repository at www.jacionline.org.

PBMCs were resuspended in PBS at a concentration of 5 to 10 × 10^6^ cells/mL and labeled with 0.5 μmol/L carboxyfluorescein succinimidyl ester (CFSE; Molecular Probes, Eugene, Ore), as described previously^33^ and in the Methods section in this article's Online Repository, to analyze the *ex vivo* activation of T and B cells. Proliferation of B and T cells was assessed by measuring CFSE dilution in combination with the same mAbs used for immunophenotyping. Analysis of cells was performed with a FACSCanto II flow cytometer (BD Biosciences) and FlowJo software (TreeStar, Ashland, Ore). Patient samples were analyzed simultaneously with PBMCs from healthy control subjects.

### ELISA

Secretion of immunoglobulins by mature B cells was assessed by testing supernatants for secreted IgM, IgG, and IgA with an in-house ELISA using polyclonal rabbit anti-human IgM, IgG, and IgA reagents and a serum protein calibrator, all from Dako (Glostrup, Denmark), as described previously.^33^

### SDS-PAGE and Western blot analysis

Blood was separated into neutrophils and PBMCs. Neutrophils (5 × 10^6^) were used for protein lysates, separated by means of SDS-PAGE, and transferred onto a nitrocellulose membrane. Individual proteins were detected with antibodies against NF-κB p50 (mouse mAb E-10; Santa Cruz Biotechnology, Dallas, Tex), against IκBα (rabbit polyclonal antiserum C-21; Santa Cruz Biotechnology), and against human glyceraldehyde-3-phosphate dehydrogenase (mouse mAb; Merck Millipore, Darmstadt, Germany).

Secondary antibodies were either goat anti-mouse-IgG IRDye 800CW, goat anti-rabbit IgG IRDye 680CW or goat anti-mouse IgG IRDye 680LT (LI-COR Biosciences, Lincoln, Neb). Relative fluorescence quantification of bound secondary antibodies was performed on an Odyssey Infrared Imaging system (LI-COR Biosciences) and normalized to glyceraldehyde-3-phosphate dehydrogenase.

### NF-κB1 protein structure

A previously resolved crystal structure of the p50 homodimer (A43-K353) bound to DNA was used to gain structural information on the NF-κB1 RHD.^34^ Ankyrin repeats of NF-κB1 (Q498-D802) were modeled by using comparative homology modeling (Modeller 9v16) with the ankyrin repeats crystal structure of NF-κB2 as a template.^35^, ^36^ There is no structural information on the region between the sixth and seventh ankyrin repeats (F751-V771),^36^ and therefore these were omitted in the model.

### Statistical analysis of lymphocyte data

Differences between groups with 1 variable were calculated with a nonpaired Student *t* test or 1-way ANOVA with the Bonferroni *post hoc* test, differences between groups with 2 or more variables were calculated with 2-way ANOVA with the Bonferroni *post hoc* test by using GraphPad Prism 6 software (GraphPad Software, La Jolla, Calif). A *P* value of less than .05 was considered significant.

## Results

### Pathogenic variants in *NFKB1* are the most common monogenic cause of CVID

**Fig 1:**
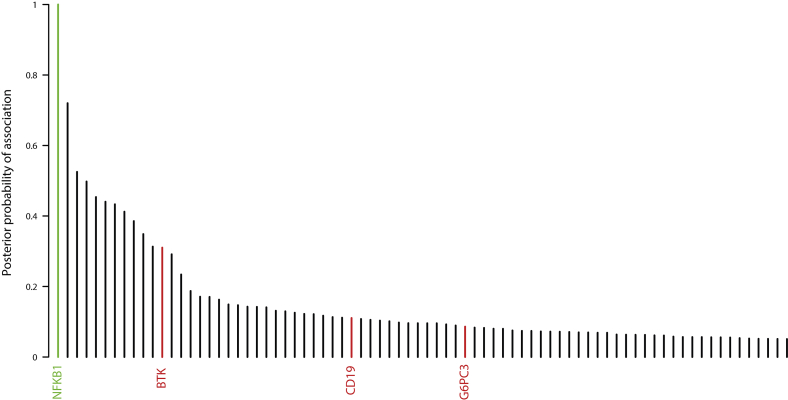
Overall BeviMed results showing that *NFKB1* has the highest posterior probability of association with disease in the NIHRBR-RD PID cohort. Genes with variants previously reported to cause PIDs are highlighted in red. Genes with posterior probabilities of greater than .05 are shown.

**Fig 2:**
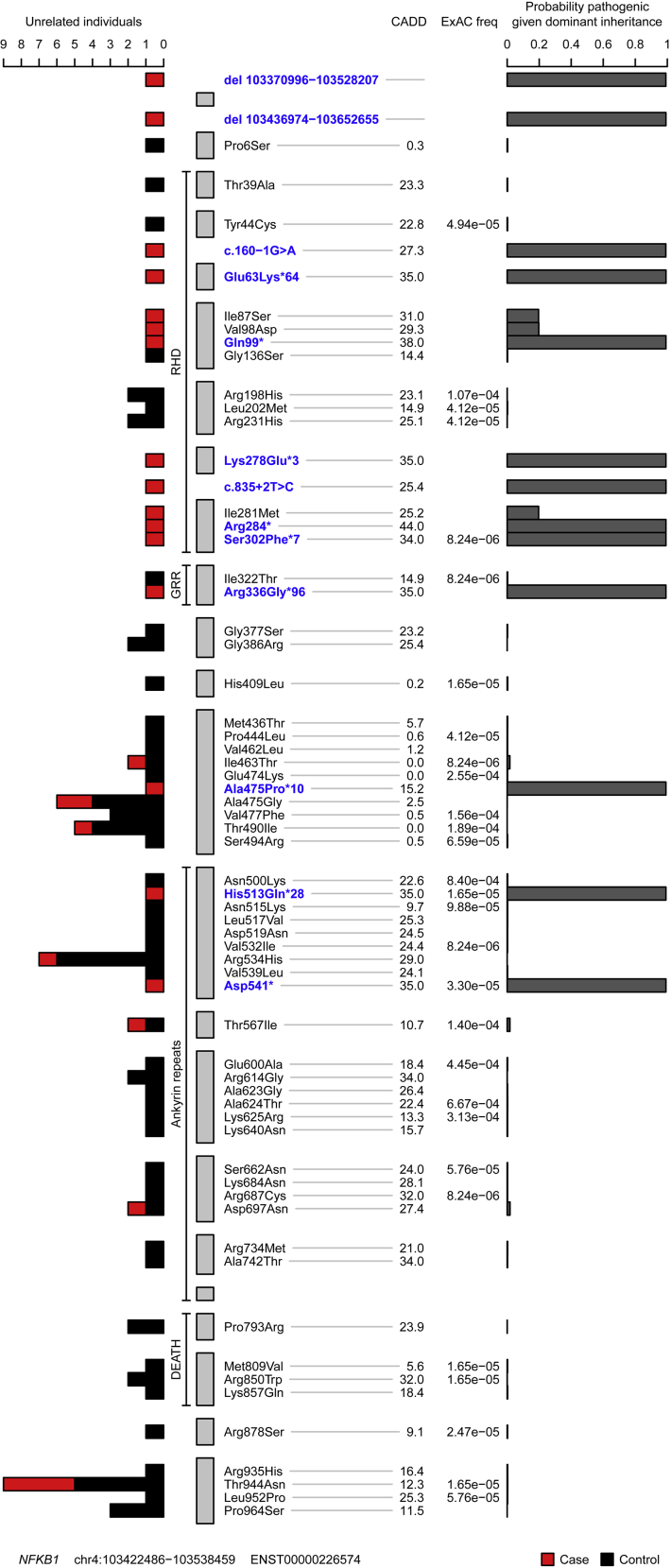
Plot of rare missense, truncating, and gene deletion *NFKB1* variants identified in the NIHRBR-RD genomes of unrelated subjects and their location relative to *NFKB1* domains. Tracks from left to right show the following: number of unrelated case (red) and control (black) subjects in whom each variant was observed; the 4 major *NFKB1* domains; each exon in transcript ENST00000226574 *(gray bars)*; variant annotation relative to transcript ENST00000226574 and genomic location of large deletions, with VEP high-effect variants and large deletions highlighted in blue; Combined Annotation Dependent Depletion *(CADD)* scores of all nonsense, frameshift, splice and missense variants; Exome Aggregation Consortium *(ExAC)* allele frequencies; and conditional probability of variant pathogenicity inferred by using BeviMed. Only variants labeled as being of moderate or high effect relative to the canonical transcript ENST00000226574 are shown. The initial inference that formed part of the genome-wide analysis included variant chr4:103423325G>A, which was observed in 1 control sample. This variant is intronic (low effect) relative to ENST00000226574 but is a splice variant (high effect) relative to the minor transcript ENST00000505458. Because variants were filtered based on the highest-effect variant annotation against any Ensembl transcript, this variant was originally included in the inference. For this plot, the inference was rerun, including only missense, truncating, and gene deletion variants relative to the canonical transcript.

In an unbiased approach to analysis, we obtained BeviMed posterior probabilities of association with PID for every individual gene in all 848 unrelated patients with PID in the NIHRBR-RD study. Genes with posterior probabilities of greater than .05 are shown in Fig 1, showing that *NFKB1* has the strongest prediction of association with disease status (1.000). All 13 high-effect variants (large deletion, nonsense, frameshift, and splice site variants) in *NFKB1* were observed in cases only, resulting in the very high posterior probabilities of pathogenicity (mean, 0.99) for this class of variants (Fig 2). On the other hand, moderate-effect variants (missense substitutions) were observed both in cases and control subjects. The majority had near-zero probability of pathogenicity, but 3 substitutions were observed in the patients with PID only and had posterior probabilities of greater than 0.15 (Fig 2), suggesting their potential involvement in the disease*.* Genomic variants with a high Combined Annotation Dependent Depletion score were found within both the PID and control cohorts, suggesting that this commonly used metric of variant deleteriousness cannot reliably distinguish disease-causing from benign variants in *NFKB1*. All 16 predicted likely pathogenic variants were private to the PID cohort, and further investigation revealed that all 16 subjects were within the diagnostic criteria of CVID (Table I).

**Table I:** Summary of the CVID patients' clinical presentation and *NFKB1* variants

Case ID	Sporadic/familial	Infections	Autoimmunity	Malignancy	Chromosome 4 position (GRCh37)	Nucleotide change	Type of variant	cDNA (NM_003998.3); Amino acid
A	Familial	●			103504037	C>T	Nonsense	c.850C>T;Arg284*
B	Familial	●	●	○	103518717	delCATGC	Frameshift	c.1539_1543del; His513Glnfs*28
C	Familial	●	●	●○	103459014	G>A	Splice acceptor	c.160-1G>A;?
D	Familial	●	●		103518801	delGA	Nonsense	c.1621_1622del; Asp541*
E	Sporadic	●	●	○	103504030	C>G	Missense	c.843C>G; Ile281Met
F	Sporadic	●		●	103488178	T>A	Missense	c.293T>A; Val98Asp
G	Sporadic	●			103488145	T>G	Missense	c.260T>G; Ile87Ser
H	Familial	●	●		103501798	T>C	Splice donor	c.835+2T>C;?
I	Sporadic	●			103370996-103528207	—	Large deletion	—
J	Familial	●			103517415	delG	Frameshift	c.1423del; Ala475Profs*10
K	Sporadic	●	●	●	103436974-103652655	—	Large deletion	—
L	Familial	●	●		103459041	delG	Frameshift	c.187del; Glu63Lysfs*64
M	Sporadic	●	●		103501790	insA	Frameshift	c.830dup; Lys278Glufs*3
N	Sporadic	●	●	●	103504086	insT	Frameshift	c.904dup; Ser302Phefs*7
O	Sporadic	●			103488180	C>T	Nonsense	c.295C>T; Gln99*
P	Sporadic	●	●		103505914	delG	Frameshift	c.1005del; Arg336Glyfs*96

●, Presence of symptoms in index patient; ○, presence of symptoms in family member of index patient.

**Fig E2:**
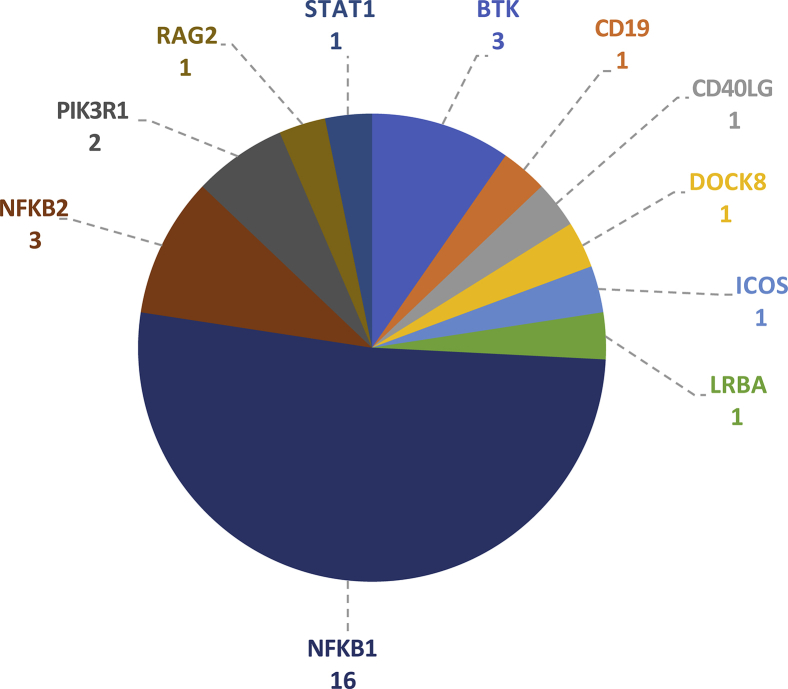


Assessment of all 390 CVID cases in our cohort for pathogenic variants showed that the next most commonly implicated genes are *NKFB2* and Bruton tyrosine kinase *(BTK)*, with 3 explained cases each (see Fig E2 in this article's Online Repository at www.jacionline.org). Importantly, based on the gnomAD data set of 135,000 predominantly healthy subjects, none of the *NFKB1* variants reported here are observed in a single gnomAD subject, even though 90% of our CVID cohort and all of the *NFKB1*-positive cases had European ancestry. Therefore our results suggest that LOF variants in *NFKB1* are the most commonly identified monogenic cause of CVID in the European population, with 16 of 390 patients with CVID explaining up to 4.1% of our cohort. None of the variants identified here had been reported in the previously described *NFKB1* cases.^6^, ^7^, ^8^, ^9^, ^10^, ^11^

**Fig 3:**
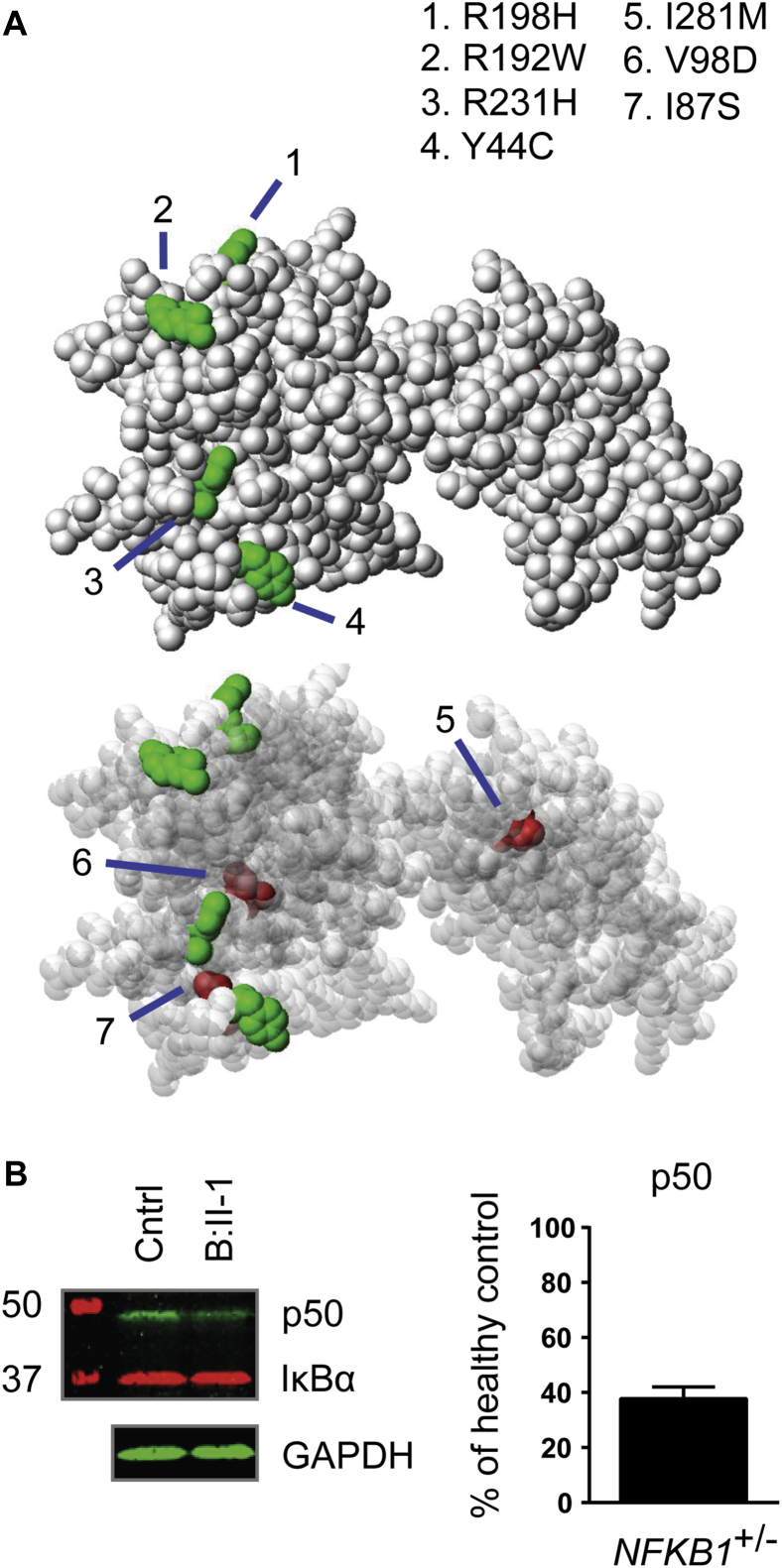
*NFKB1* LOF variants lead to haploinsufficiency of the p50 protein. **A,** Localization of RHD substitutions with a high Combined Annotation Dependent Depletion *(CADD)* score (>20) within the structure of the NF-κB p50 monomer. Shown is a solid *(top panel)* and a transparent *(bottom panel)* sphere representation of the NF-κB p50 monomer. Perturbed residues indicated in green were observed in a control data set and are located on the outside of the structure, whereas residues shown in red were perturbed exclusively in the PID cohort and are buried inside the structure. **B,** Western blot analysis targeting p50, IκBα, and glyceraldehyde-3-phosphate dehydrogenase *(GAPDH)* of *NFKB1* variant carriers. *Left*, Representative blot of a healthy control subject and patient B-II:1; *right*, summary of 16 *NFKB1* variant carriers showing haploinsufficiency expressed as a percentage of healthy control subjects on the same blot corrected for GAPDH (mean ± SEM).

**Fig E3:**
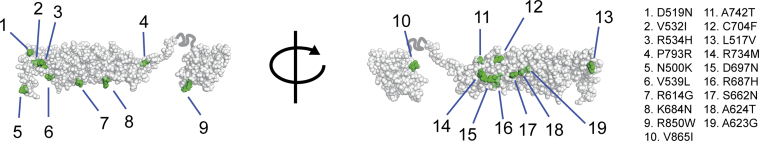


The *NFKB1* gene encodes the p105 protein, which is processed to produce the active DNA-binding p50 subunit.^13^ The 16 potentially pathogenic variants we identified were all located in the N-terminal p50 part of the protein (Fig 2). The effects of the 3 rare substitutions on NF-κB1 structure were less clear than those of the truncating and gene deletion variants, and therefore we assessed their position in the crystal structure of the p50 protein. Their location in the inner core of the RHD (Fig 3, *A*) suggested a potential effect on protein stability, whereas other rare substitutions in the NIHRBR-RD cohort were found in locations less likely to affect this (Figs 2 and 3, *A*, and see Fig E3 in this article's Online Repository at www.jacionline.org).

### *NFKB1* LOF as the disease mechanism

**Fig E4:**
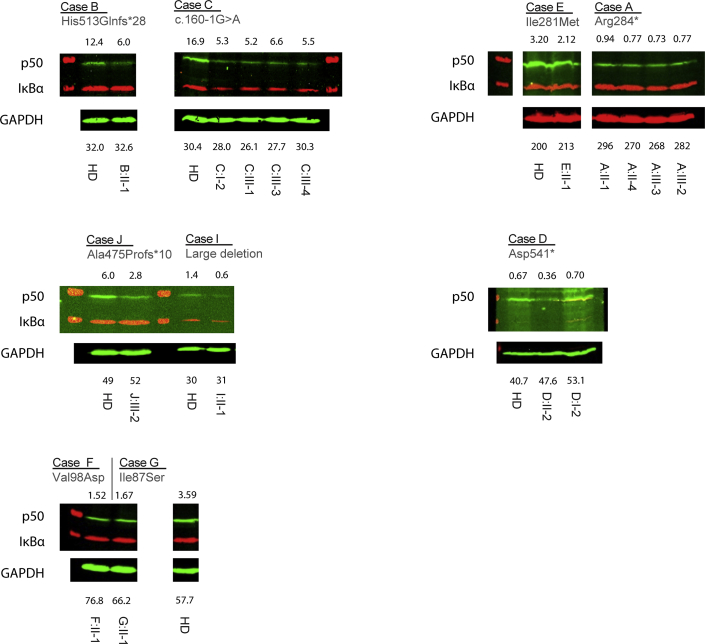


Twelve patients with truncating variants (Arg284*, His513Glnfs*28, c.160-1G>A, and Asp451*), 1 patient with gene deletion (del 103370996-103528207), and 3 patients with putative protein destabilizing missense variants (Ile281Met, Val98Asp, and Ile87Ser) were investigated for evidence of reduced protein level. Assessment of the NF-κB1 protein level in PBMCs or neutrophils in 9 index cases and 7 *NFKB1* variant–carrying relatives demonstrated a reduction in all subjects (Fig 3, *B*, and see Fig E4 in this article's Online Repository at www.jacionline.org). Relative fluorescence quantification of the bands confirmed this and demonstrated a protein level of 38% ± 4.3% (mean ± SEM) compared with healthy control subjects. There was no difference between clinically affected and clinically unaffected subjects (36% ± 4.4% vs 42% ± 10.1%, n = 11 vs n = 5, *P* = .50). Our observations indicate that the pathogenic *NFKB1* variants result in LOF of the NF-κB1 p50 subunit because reduction in protein levels was seen in all carriers regardless of their clinical phenotype and was absent in family members who were noncarriers.

### Variable disease manifestations in *NFKB1* LOF subjects

**Fig 4:**
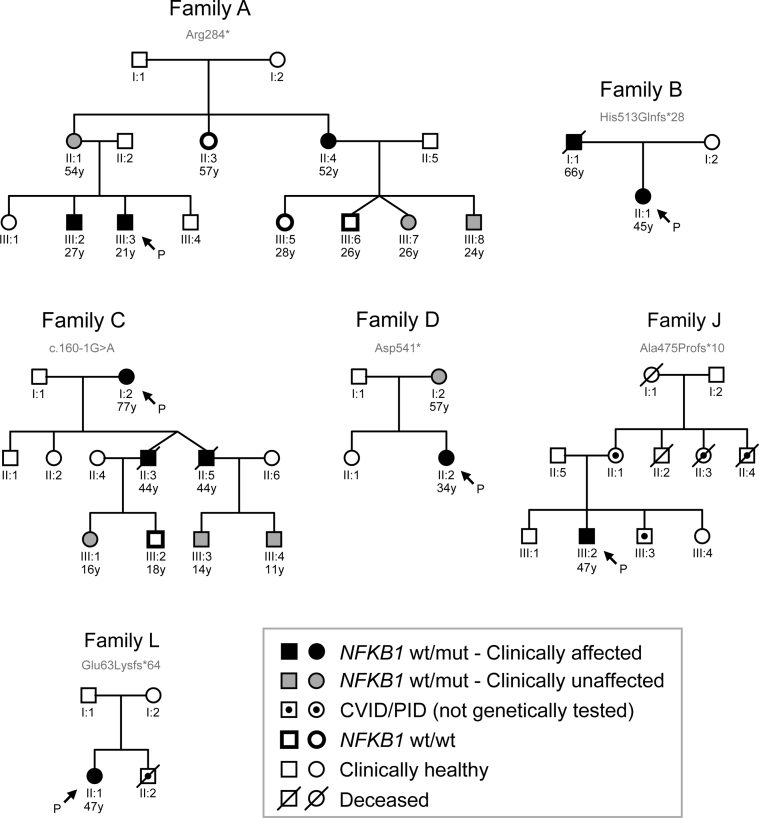
Pedigrees of familial *NFKB1* cases. Six affected families for which pedigree information and additional family members were available. *P*, Proband/index cases.

**Fig E5:**
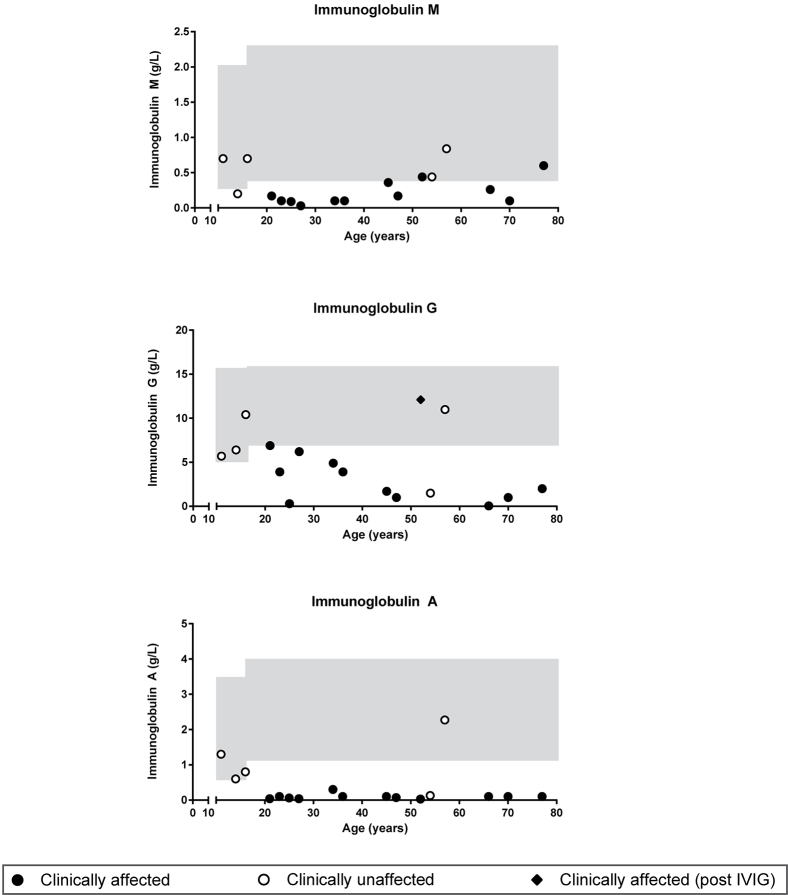


Seven subjects had evidence of familial disease (Table I), prompting us to investigate genotype-phenotype cosegregation and disease penetrance in cases for which pedigree information and additional family members were available (Fig 4 and see Tables E1 to E3 in this article's Online Repository at www.jacionline.org). The age at which hypogammaglobulinemia becomes clinically overt is highly variable (see Fig E5 in this article's Online Repository at www.jacionline.org), as shown by pedigree C in which grandchildren carrying the c.160-1G>A splice-site variant had IgG subclass deficiency (C:III-3 and C:III-4), in one case combined with an IgA deficiency (C:III-3). Although not yet overtly immunodeficient, the clinical courses of their fathers (C:II-3 and C:II-5) and grandmother (C:I-2) predict this potential outcome and warrant long-term clinical follow-up of these children.

We also observed variants in subjects who were clinically asymptomatic. Pedigree A highlights variable disease penetrance: the healthy mother (A:II-1) carries the same Arg284* variant as 2 of her clinically affected children (A:III-2 and A:III-3). Identification of this nonsense variant prompted clinical assessment of the extended kindred and demonstrated that her sister (A:II-4) had recurrent sinopulmonary disease and nasal polyps with serum hypogammaglobulinemia consistent with a CVID diagnosis. Overall, based on the clinical symptoms observed at the time of this study across 6 pedigrees, the penetrance of *NFKB1* variants with respect to the clinical manifestation of CVID is incomplete (about 60% in our cohort, 11 affected subjects among 18 variant carriers), with varied expressivity not only of age at disease onset but also of specific disease manifestations, even within the same pedigree.

The clinical disease observed among the *NFKB1* variant carriers is characteristic of progressive antibody deficiency associated with recurrent sinopulmonary infections (100% of clinically affected subjects) by encapsulated microbes, such as *Streptococcus pneumoniae* and *Haemophilus influenzae* (see Table E1). The clinical spectrum of *NFKB1* LOF includes massive lymphadenopathy (24%), unexplained splenomegaly (48%), and autoimmune disease (48%), either organ-specific and/or hematologic in nature (mainly autoimmune hemolytic anemia and idiopathic thrombocytopenic purpura, see Tables E1 and E2). The percentage of autoimmune complications is based on the presence of autoimmune cytopenias (autoimmune hemolytic anemia, idiopathic thrombocytopenic purpura [<50-75 × 10^6^/mL], autoimmune neutropenia, and Evans syndrome), alopecia areata/totalis, vitiligo, and Hashimoto thyroiditis among the clinically affected cases. Granulomatous-lymphocytic interstitial lung disease and splenomegaly were considered lymphoproliferation. Enteropathy, liver disease, colitis, and a mild decrease in platelet count (>100 × 10^6^/mL) were neither included in those calculations nor scored separately. Histologic assessment of liver disease found in 3 patients showed no evidence of autoimmune or granulomatous liver disease, although fibrosis and cirrhosis were observed in these male patients. Finally, the number of oncological manifestations, predominantly hematologic, was noticeable. There were 2 cases with solid tumors (parathyroid adenoma and breast cancer) and 4 cases with hematologic malignancies (B-cell non-Hodgkin lymphoma, diffuse large B-cell lymphoma, follicular lymphoma, and peripheral T-cell lymphoma), which add up to 6 (28.6%) of 21 cases.

### B-cell phenotype in subjects with *NFKB1* LOF mutations and immune cell activation

**Fig 5:**
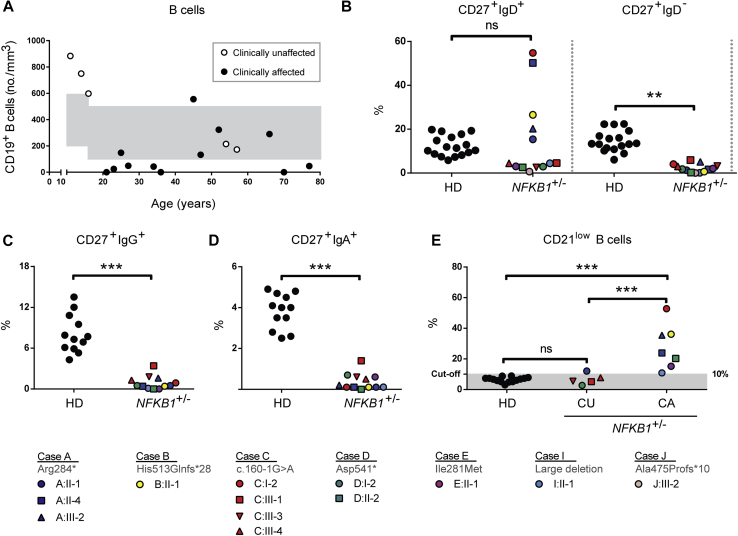
Decreased class-switched memory B-cell and increased CD21^low^ B-cell counts in *NFKB1* LOF variant carriers. **A,** Absolute numbers of CD19^+^ B cells; each *dot* represents a single subject and his or her age. Age-dependent reference values are shown in gray. **B-E,** Percentages within CD19^+^CD20^+^ B lymphocytes of CD27^+^IgD^+^ (nonswitched memory or marginal-zone B cells) and CD27^+^IgD^−^ (switched memory B cells; Fig 5, *B*) or CD27^+^IgG^+^ (Fig 5, *C*), CD27^+^IgA^+^ (Fig 5, *D*), or CD21^low^CD38^low/dim^ (Fig 5, *E*). *CA*, Clinically affected subjects with an LOF variant in *NFKB1*; *CU*, clinically unaffected subjects with an LOF variant in *NFKB1*; *HD*, healthy donor. The gating strategy is shown in Fig E6, *A*. Only subjects with sufficient B cells could be analyzed. *P* values were determined by using 1-way (Fig 5, *E*) or 2-way (Fig 5, *B*) ANOVA with the Bonferroni *post hoc* test or unpaired Student *t* test (Fig 5, *C* and *D*). *ns*, Not significant. **P ≤ .01 and ***P ≤ .001.

**Fig 6:**
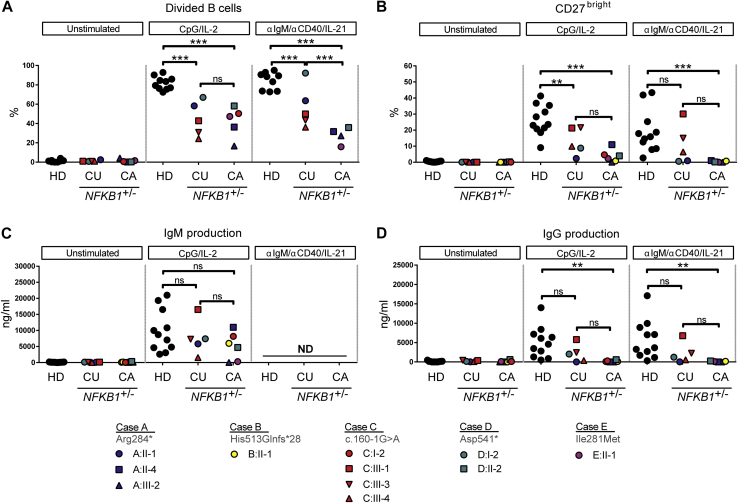
*Ex vivo* class-switch recombination defect of subjects carrying *NFKB1* LOF variants is linked to the more extreme phenotype. Six-day culture of CFSE-labeled lymphocytes normalized for B-cell numbers: unstimulated, CpG/IL-2 stimulated (T cell–independent activation), or anti-IgM/anti-CD40/IL-21 stimulated (T cell–dependent activation). **A,** Percentage of divided B cells, as measured based on CFSE dilution. **B,** Percentage of CD27^++^ plasmablasts. The gating strategy is shown in Fig E7, *A*. **C** and **D,** IgM and IgG production in supernatants of 6-day cultures. *CA*, Clinically affected subjects with an LOF variant in *NFKB1*; *CU*, clinically unaffected subject; *HD*, healthy donor. Only subjects with sufficient B cells could be analyzed. *P* values were determined by using 2-way ANOVA with the Bonferroni *post hoc* test. *ns*, Not significant. **P ≤ .01 and ***P ≤ .001.

**Fig E6:**
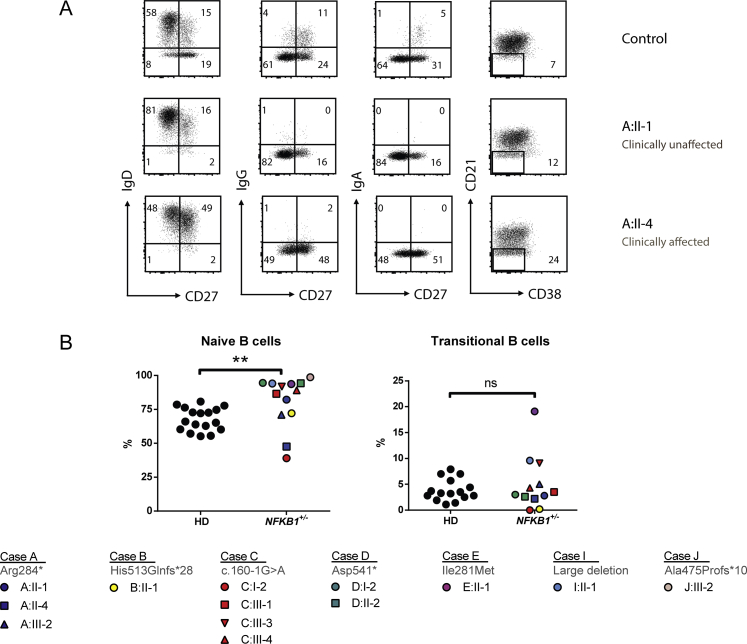


**Fig E7:**
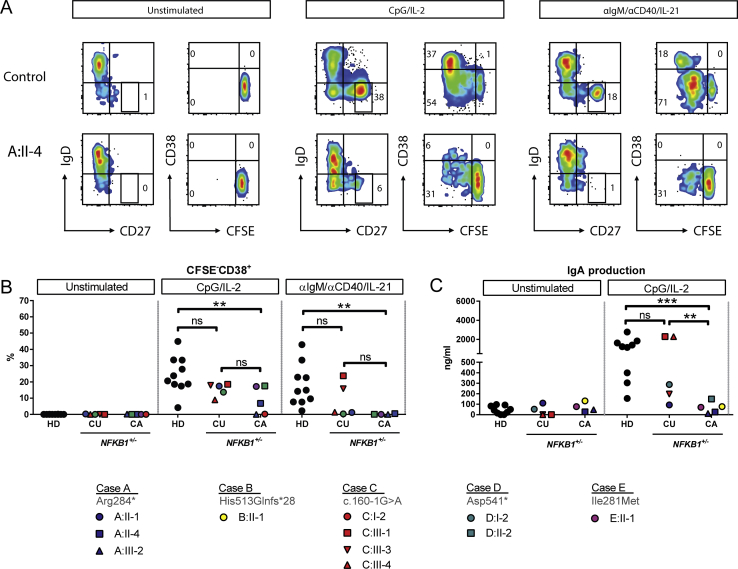


Index cases and family members carrying *NFKB1* variants were approached for repeat venipuncture for further functional assessment. In clinically affected subjects, B-cell numbers and phenotypes were indistinguishable from those described for patients with CVID (Fig 5 and see Fig E6 in this article's Online Repository at www.jacionline.org).^37^ However, in clinically unaffected subjects the absolute B-cell count was often normal or increased (Fig 5, *A*). In all subjects with *NFKB1* LOF variants, the numbers of switched memory B cells were reduced (Fig 5, *B-D*), whereas a broad range of nonswitched memory B cells was observed. This demonstrates that although the clinical phenotype of *NFKB1* LOF variants is partially penetrant, all carriers have a deficiency in class-switched memory B-cell generation. The presence of increased numbers of the CD21^low^ population described in patients with CVID discriminates between clinically affected and unaffected subjects with *NFKB1* LOF variants (Fig 5, *E*). B cells from subjects with *NFKB1* LOF variants demonstrated impaired proliferative responses to anti-IgM/anti-CD40/IL-21 and CpG/IL-2 (Fig 6, *A*); this corresponded with the inability to generate plasmablasts (CD38^+^/CD27^++^), which was most pronounced in the more extreme phenotypes (ie, clinically affected cases; Fig 6, *B*, and see Fig E7, *B*, in this article's Online Repository at www.jacionline.org). Similarly, *ex vivo* IgG production was reduced in subjects with LOF variants, whereas IgM levels in the supernatants were normal (Fig 6, *C* and *D*, and see Fig E7, *C*), which is compatible with hypogammaglobulinemia.

### T-cell phenotype in subjects *NFKB1* LOF variants

**Fig E8:**
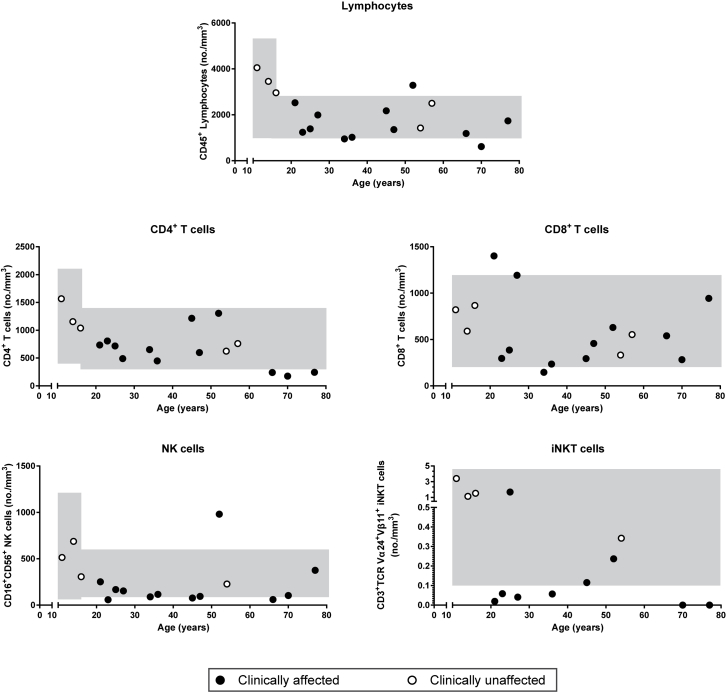


**Fig E9:**
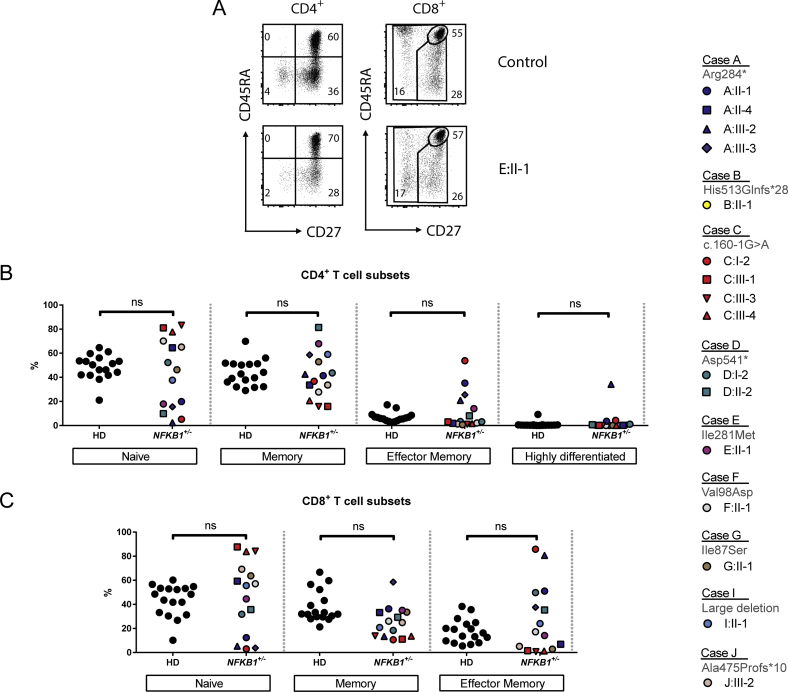


**Fig E10:**
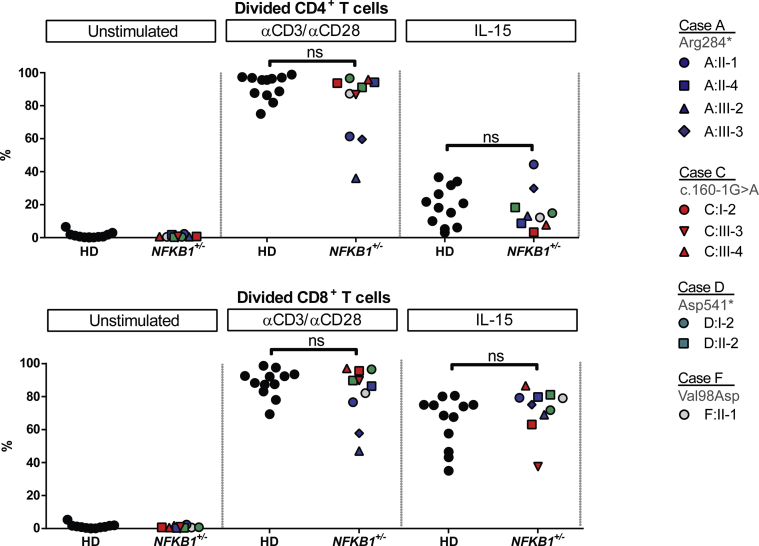


The T-cell phenotype was largely normal in the subset distribution (see Figs E8 and E9 in this article's Online Repository at www.jacionline.org). Similar to the knockout mouse model,^38^ we found an aberrant number of invariant natural killer T cells in clinically affected subjects (see Fig E8). T-cell proliferation was intact on anti-CD3/anti-CD28 or IL-15 activation (see Fig E10 in this article's Online Repository at www.jacionline.org). Because invariant natural killer T cells have been implicated in diverse immune reactions,^39^ this deficiency might contribute to the residual disease burden in immunoglobulin replacement–treated patients, some of whom had acute or chronic relapsing infection with herpes virus and, in one case, JC virus.

## Discussion

In our study we show that LOF variants in *NFKB1* are present in 4% of our cohort of patients with CVID, being the most commonly identified genetic cause of CVID. Furthermore, we highlight specific features of these patients that distinguish them within the diagnostic category of CVID, which otherwise applies to an indiscrete phenotype acquired over time that is termed common and variable. The majority of the genetic variants we report here truncate or delete 1 copy of the gene; together with pedigree cosegregation analyses, protein expression, and B-cell functional data, we conclude that *NFKB1* LOF variants cause autosomal dominant haploinsufficiency. This has now been recognized as the genetic mode of inheritance for at least 17 known PIDs, including those associated with previously reported variants in *NFKB1*.^6^, ^40^, ^41^, ^42^ In monogenic causes of PID, incomplete penetrance has been more frequently described in haploinsufficient relative to dominant negative PID disease, having been reported in more than half of the monogenic autosomal dominant haploinsufficient immunologic conditions described.^40^ This might be because dominant negative gain-of-function mutations cause disease by expression of an abnormal protein at any level, whereas, as seen in this study, haploinsufficiency is predicted to lead to 50% residual function of the gene product. By definition, incomplete penetrance of a genetic illness will be associated with substantial variation in the clinical spectrum of disease, and the spectrum seen in this study is consistent with prior reports; in 3 pedigrees with 20 subjects^6^ harboring heterozygous mutant *NFKB1* alleles, the age of onset varied from 2 to 64 years, with a high variety of disease severities, including 2 mutation carriers who were completely healthy at the ages of 2 and 43 years.

It is important to temper skepticism of partial penetrance of immune genetic lesions with our knowledge that individual immune genes might have evolved in response to selection pressure for host protection against specific pathogens.^43^ Consequently, within the relatively pathogen-free environment of developed countries, the relevant pathogen for triggering disease might be scarce, and reports documenting partial penetrance of the clinical phenotype will increase. This makes the traditional approaches to genetics for determining causality difficult. The BeviMed algorithm used in this study prioritized both the gene *NFKB1* and individual variants within *NFKB1* for contribution to causality; the power of methods like this will increase with greater data availability. Identification of a number of rare *NFKB1* variants with high Combined Annotation Dependent Depletion scores in both the PID and control data sets highlights the potential for false attribution of disease causality when the genetics of an individual case are considered outside the context of relevant control data.

Currently healthy family members carrying the same *NFKB1* LOF variant demonstrated similar reductions in p50 expression and low numbers of switched memory B cells as their relatives with CVID. The longitudinal research investigation of these subjects could help identify the additional modifiers, including epigenetic or environmental factors, that influence the clinical penetrance of these genetic lesions. The similarity of results seen in patients with large heterozygous gene deletions and in those with more discrete substitutions is consistent with haploinsufficiency as the shared disease mechanism.

In patients with mild antibody deficiency, it is often difficult to decide when to initiate replacement immunoglobulin therapy; this might be the case for subjects and their family members identified with LOF *NFKB1* variants. Two measures seem to correlate well with clinical disease.

First, the class-switch defect and lower IgG and IgA production *ex vivo* was examined. Immunoglobulin class-switching is known to be regulated by NF-κB. Mutations in NF-κB essential modulator cause a class-switch–defective hyper-IgM syndrome in human subjects,^20^ as well as in p50 knockout mice.^13^, ^44^, ^45^ Haploinsufficiency of NF-κB might result in defective class-switch recombination because of poor expression of activation-induced cytidine deaminase, a gene regulated by NF-κB, which, when absent, is also associated with immunodeficiency.^46^

Second, the ability to measure the CD21^low^ B-cell population is widespread in diagnostic immunology laboratories, and our study identifies this marker to correlate with NF-κB disease activity. Although the function of these cells remain to be fully elucidated,^47^ this laboratory test might be useful for longitudinal assessment of clinically unaffected subjects identified with LOF *NFKB1* variants.

Apart from having recurrent and severe infections (including viral disease) for which these patients had been given a diagnosis of PID in the first place, autoimmunity and unexplained splenomegaly are very common manifestations in our patient cohort similar to the other heterozygous *NFKB1* cases described.^6^, ^7^, ^8^, ^9^, ^10^, ^11^ Although autoimmunity has been subject to variable percentages per cohort study,^3^, ^48^, ^49^ it seems that these complications occur more frequently in *NFKB1*-haploinsufficient patients compared with unselected CVID cohorts. In contrast to IKAROS defects but similar to cytotoxic T lymphocyte–associated protein 4 *(CTLA4)* haploinsufficiency, we observed that *NFKB1* haploinsufficiency can also result in chronic and severe viral disease, as noted for cytomegalovirus and JC virus infections in 3 of our patients. In the study of Maffucci et al,^11^ one of the *NFKB1*-affected patients also experienced *Pneumocystis jirovecii* infection and progressive multifocal leukoencephalopathy (PML), which is suggestive for JC virus infection. Whether the B-cell defect in *NFKB1* haploinsufficiency is responsible for these nonbacterial infections is unclear.^50^, ^51^ PML is most often discovered in the context of an immune reconstitution inflammatory syndrome, as seen in patients with HIV receiving antiretroviral therapy and in patients with multiple sclerosis after natalizumab discontinuation.^52^ Although the exact contribution of B-cell depletion in PML pathogenesis is unknown, the increased PML risk in rituximab-treated patients^53^ suggests a protective role for B cells.

Three subjects in this cohort had liver failure, and an additional 3 had transaminitis. Although autoimmunity is suspected, a nonhematopoietic origin of liver disease cannot be excluded in the absence of autoantibodies and nodular regenerative disease. Mouse models have suggested a nonimmune role for NF-κB signaling in patients with liver failure.^13^, ^54^, ^55^, ^56^

In the cohort of patients with *NFKB1* variants, we identified a number of malignancies. Malignancies in patients with PIDs have been cited as the second-leading cause of death after infection,^57^, ^58^ and murine models have demonstrated that haploinsufficiency of NF-κB1 is a risk factor for hematologic malignancy.^59^ In a large CVID registry study of 2212 patients, 9% had malignancies, with one third being lymphomas, some presenting before their CVID diagnosis.^49^ Despite the fact that our cohort is relatively small, we found oncologic manifestations in 29% of our cases (two thirds being lymphoma), suggesting that malignancies in patients with *NFKB1* haploinsufficiency can occur more often than in unselected patients with CVID. In a study in 176 patients with CVID, among the 626 relatives of patients with CVID, no increase in cancer risk was observed,^60^ suggesting that when this does occur, as in this study (3/7), it might be due to a shared genetic lesion. Therefore in a pedigree with an LOF variant in *NFKB1*, any relatives with cancer should be suspected of sharing the same pathogenic variant.

In conclusion, previous publications^61^, ^62^ have suggested that CVID is largely a polygenic disease. Our results provide further evidence that LOF variants in *NFKB1* are the most common monogenic cause of disease to date, even in seemingly sporadic cases. In these patients there is a clear association with complications, such as malignancy, autoimmunity, and severe nonimmune liver disease; this is important because the excess mortality seen in patients with CVID occurs in this group.^48^ The screening for defined pathogenic *NFKB1* variants accompanied by B-cell phenotype assessment has prognostic value and is effective in stratifying these patients.

**Key messages**

• Pathogenic variants in *NFKB1* are currently the most common known monogenic cause of CVID.
• There is a clear association with complications, such as autoimmunity and malignancy, features associated with worse prognosis.
• These patients can be stratified by *NFKB1* protein level and B-cell phenotype.

## Supplementary data

Methods

Tables E1-E3

Legends for Figures E1-E10
