# Supplementary material for: Loss-of-function nuclear factor κB subunit 1 (NFKB1) variants are the most common monogenic cause of common variable immunodeficiency in Europeans
Source: J Allergy Clin Immunol. 2018 Oct;142(4):1285–96. doi: 10.1016/j.jaci.2018.01.039 (PMC6148345; doi:10.1016/j.jaci.2018.01.039)
Supplement: Methods [file mmc1.doc]

**Supplemental Methods**

**Immunophenotyping**

Peripheral blood mononuclear cells (PBMCs) were isolated using standard density gradient centrifugation techniques using Lymphoprep (Nycomed, Oslo, Norway). Absolute numbers of lymphocytes, T cells, B cells and NK cells were determined with Multitest six-color reagents (BD Biosciences, San Jose, USA), according to manufacturer’s instructions. For the immunophenotyping the PBMCs were resuspended in PBS, containing 0.5% (w/v) BSA and 0.01% sodium azide and incubated with saturating concentrations of fluorescently labeled conjugated monoclonal antibodies. Analysis of cells was performed using a FACSCanto-II flowcytometer and FlowJo software. Patient samples were analyzed simultaneously with PBMCs from healthy controls. The following directly conjugated monoclonal antibodies were used: CD4 PE-Cy7 [348809], CD8 PerCP-Cy5.5 [341050], CD20 PerCP-Cy5.5 [332781], CD21 FITC [561372], CD27 APC [337169], CD38 PE-Cy7 [335825], CD45 APC-Cy7 [348815], IgD PE [555779], and TCRγδ FITC [347903] from BD (San Jose, USA), CD3 Alexa 700 [56-0038-41], CD19 Alexa 700 [56-0199-42] and CD27 APC-eFluor 780 [47-0279-42] from eBioscience (San Diego, USA), CD24 FITC [M1605] and CD27 FITC [M1764] from Sanquin (Amsterdam, the Netherlands), CD45RA (2H4-RD1) PE [6603181], TCRαβ PE [PN A39499], TCR Vα24 FITC [PM IM1589] and TCR Vβ11 PE [PN IM2290] from Beckman Coulter (Brea, USA), IgM FITC [F0317] and IgG FITC [F0158] from Dako (Glostrup, Denmark), IgA FITC [130-093-071] from Miltenyi Biotec (Bergisch Gladbach, Germany).

**B and T cell functional assay**

To analyze the *ex vivo* activation of T and B cells, PBMCs were resuspended in PBS at a concentration of 5–10×10^6^ cells/ml and labeled with 0.5μM CFSE (Molecular Probes) in PBS for 10 minutes at 37°C under constant agitation. Cells were washed and subsequently resuspended in Iscove’s Modified Dulbecco’s medium (IMDM) supplemented with 10% fetal calf serum (BioWhittaker), antibiotics, and 3.57×10^–4^%(v/v) β-mercaptoethanol (Merck). Labeled PBMCs containing a fixed number of 2×10^4^ (96-well plate) or 10×10^4^ B cells (48-well plate) per well in a flat-bottom plate for 6 days at 37°C and stimulated with saturating amounts of anti-IgM mAb (clone MH15; Sanquin), anti-CD40 mAb (clone 14G7; Sanquin) and 20ng/ml IL-21 (Invitrogen), or 1μg/ml CpG oligodeoxynucleotide 2006 (Invivogen) and 100U/ml IL-2 (R&D Systems), or anti-CD3 (clone 1xE) and anti-CD28 (clone 15E8), or IL-15 (R&D Systems). Proliferation of B and T cells was assessed by measuring CFSE dilution in combination with the same mAbs used for immunophenotyping and analyzed using a FACSCanto-II flowcytometer and FlowJo software.
