# Supplementary material for: Loss-of-function nuclear factor κB subunit 1 (NFKB1) variants are the most common monogenic cause of common variable immunodeficiency in Europeans
Source: J Allergy Clin Immunol. 2018 Oct;142(4):1285–96. doi: 10.1016/j.jaci.2018.01.039 (PMC6148345; doi:10.1016/j.jaci.2018.01.039)
Supplement: Tables E1-E3 [file mmc2.doc]

| **Table E1. Clinical data of individuals carrying *NFKB1* variants** | | | | | | | | |
| --- | --- | --- | --- | --- | --- | --- | --- | --- |
| **Case ID** | **Year of birth** | **Age at PID diagnosis** | **Clinical diagnosis** | **Major symptoms at onset** | **Infections** | **Autoimmunity / autoinflammation** | **Malignancy** | **Survival** |
| Case A | | | | | | | | |
| A:II-1 | 1961 | - | Healthy | - | - | - | - | - |
| A:II-4 | 1963 | 2015 | CVID | Sinusitis 2012  Pneumonia 2015 | *S. pneumoniae* | - | - | - |
| A:III-2 | 1989 | 1992 | XLA | - | Frequent sinopulmonary infections | - | - | - |
| A:III-3 | 1995 | 2005 | XLA | Pneumonias 2002 | EBV-related splenomegaly 2005  JC-virus 2016 | - (splenomegaly) | - | - |
| Case B | | | | | | | | |
| B:I-1 | 1939 | 1967 | CVID | Appendicitis-abscess 1965  Bacterial meningitis 1967  Pneumonias 1967 | *S. pneumoniae* | AIHA 2005 | B-NHL (EBV-negative) 2007 | Died 2007:  heart attack |
| B:II-1 | 1968 | 2011 | CVID | Pneumonias 2010 | *S. pneumoniae,*  *H. influenzae* | Alopecia areata 1988  Vitiligo 1989 Hypothyroiditis 2003 | - | - |
| Case C | | | | | | | | |
| C:I-2 | 1938 | 1990 | CVID | Sinusitis  Pneumonias  ICU respiratory failure (1978)  Bronchiectasis | *S. pneumoniae,*  *H. influenzae* | Sister and her children have autoimmune disease (MS, IDDM1 & SLE) | Parathyroid adenoma | - |
| C:II-3 | 1972 | 1988 | CVID | Pneumonias  Bronchiectasis |  | IDDM1 |  | Died in 2008:  during 2^nd^ OLT (1^st^ OLT 2005) |
| C:II-5 | 1972 | 1985 | CVID | OMAs  Oral ulcers  Sore throats | *H. influenzae*  *S. pneumoniae*  *C. albicans*  *A. fumigatus* | - | DLBCL (EBV-neg) | Died in 2011: DLBCL |
| C:III-1 | 1999 | - | Healthy | - | - | - | - | - |
| C:III-3 | 2001 | - | Healthy | - | - | - | - | - |
| C:III-4 | 2004 | - | Healthy | - | - | - | - | - |
| Case D | | | | | | | | |
| D:I-2 | 1958 | - | Healthy | - | - | Thyroid disease | - | - |
| D:II-2 | 1981 | 1999 | CVID | ITP and anemia  Hypogammaglobulinemia  Asthma | Pneumonias  Sinusitis  *E. coli*, urinary tract infections | ITP  Splenomegaly  GLILD  Periodontitis | - | - |
| Case E | | | | | | | | |
| E:II-1 | 1992 | 1999 | ALPS | Bronchiectasis  Hypogammaglobulinemia following multiple courses of Rituximab therapy | Chest infections  Sinusitis | Autoimmune neutropenia  ITP | - | - |
| Case F | | | | | | | | |
| F:II-1 | 1946 | 2000 | CVID | Severe pneumonias  Bronchiectasis  Lung fibrosis Cellulitis R leg | - | Hyperthyroidism | Follicular lymphoma (2005); recurrence (2008) | - |
| Case G | | | | | | | | |
| G:II-1 | 1980 | 2001 | CVID | Severe pneumonia  Bronchiectasis | COPD lobectomy (2009)  *M. avium* | Mild splenomegaly Chronic diarrhea | - | - |
| Case H | | | | | | | | |
| H:II-1 | 1973 | 1997 | CVID | ITP and AIHA  Splenomegaly | Pneumonia  Sinusitis  Invasive CMV | Chronic diarrhea with villous atrophy | - | Died in 2008  CMV |
| Case I | | | | | | | | |
| I:II-1 | 1991 | 2009 | CVID | Multi-dermatomal shingles (age 12)  Recurrent pneumonia  Bronchiectasis  Sinusitis | *S. pneumoniae*  *H. influenzae* | Mild thrombocytopenia | - | - |
| Case J | | | | | | | | |
| J:III-2 | 1969 | 2004 | CVID | Recurrent pneumonia  Sinusitis, otitis media  Recurrent prostatitis | *M. catarrhalis*  *H. influenzae*  *P. aeruginosa*  *S. pneumoniae*  2011 CMV DNA detected in urine | Diabetes (corticosteroid-induced) | - | - |
| Case K | | | | | | | | |
| K:II-1 | 1952 | 1996 | CVID | Recurrent pneumonias, otitis and sinusitis  Pneumococcal meningitis | *P. aeruginosa*  *H. influenzae*  *S. pneumonia*  *S. marcescens* | AIHA | Peripheral T-cell lymphoma, received CHOP | - |
| Case L | | | | | | | | |
| L:II-1 | 1969 | 1991 | CVID | Initially few symptoms – tested for IgG levels when her brother died of bacterial meningitis on background recurrent respiratory infections and low Ig’s. Respiratory infections soon became apparent. |  | AIHA 1999 (splenectomy) | - | - |
| Case M | | | | | | | | |
| M:II-1 | 1985 | 2012 | CVID | Respiratory infections, with bronchiectasis and chronic sinusitis.  Chronic diarrhea | *P. aeruginosa* (bronchi/sinuses- 2012,3,4,5,6),  *S. aureus* 2015,6, RSV 2015, atypical myco-bacterium infection 2009, *C. difficile* - year unknown, plantar warts (HPV), ongoing Herpes Zoster, disseminated 2011 (while on azathioprine) | Evans syndrome 2009 (Rituximab)  Autoimmune enteropathy 2010  Vitamin B12 deficiency (suspected pernicious anemia) | - | - |
| Case N | | | | | | | | |
| N:II-1 | 1959 | 2015 | CVID | Hypogammaglobulinemia.  Generalised lymphadenopathy and splenomegaly  Pancytopenia | EBV, CMV  Neutropenic sepsis (without positive cultures) | Alopecia totalis | Breast cancer |  |
| Case O | | | | | | | | |
| O:II-1 | 1978 | 2001 | CVID | Frequent bacterial infections  Chronic diarrhea | *Giardia lamblia* | - | - | - |
| Case P | | | | | | | | |
| P:II-1 | 1961 | 2004 | CVID | AIHA (treated with Rituximab 2012).  ITP and autoimmune neutropenia.  Recurrent sinus and respiratory tract infections | Chronic Norovirus  Rhinovirus  *H. influenzae*  *S. pneumoniae* | GLILD.  Polyarthritis (RA-like: RF/ANA negative) | - | - |

**Note:**

*H. influenzae* strains are non-typeable (= uncapsulated) unless mentioned specifically.

**Abbreviations:**

AIHA: Autoimmune haemolytic anaemia; B-NHL: B non-Hodgkin lymphoma; CVID: Common variable immunodeficiency; DLBCL: Diffuse Large B cell lymphoma; GLILD: Granulomatous-lymphocytic inflammatory lung disease; ITP: immune thrombocytopenia; OLT: Orthopic liver transplantation; XLA: X-linked agammaglobulinemia

**Table E2. Symptoms and organ involvement in individuals carrying *NFKB1* variants**

| **Case ID** | **Year of birth** | **Lung** | **Lymph nodes** | **Spleen** | **Liver function** | **Gastro-intestinal tract** | **Brain** |
| --- | --- | --- | --- | --- | --- | --- | --- |
| Case A | | | | | | | |
| A:II-1 | 1961 | - | - | - | - | - | - |
| A:II-4 | 1963 | - | - | - | - | - | - |
| A:III-2 | 1989 | - | - | - | - | - | - |
| A:III-3 | 1995 | - | Enlarged | Splenectomy for splenomegaly, suspected malignancy | Transaminitis | - | - |
| Case B | | | | | | | |
| B:I-1 | 1939 | - | - | Splenomegaly | Hepatomegaly | - | - |
| B:II-1 | 1968 | - | - | - | - | - | - |
| Case C | | | | | | | |
| C:I-2 | 1938 | Bronchiectasis  Lung fibrosis | - | - | Transaminitis | - | - |
| C:II-3 | 1972 | Bronchiectasis | Enlarged (cervical and axillary) | Enlarged | Liver fibrosis (1998) without granulomas, no signs of infection or autoimmunity | - | - |
| C:II-5 | 1972 | Bronchiectasis | - | - | Liver cirrhosis (1996), suspect of hepatitis C virus infection | Duodenal partial villous blunting, no granuloma and absence of colonic plasmacells | Tremor |
| C:III-1 | 1999 | - | - | - | - | - | - |
| C:III-3 | 2001 | - | - | - | - | - | - |
| C:III-4 | 2004 | - | - | - | - | - | - |
| Case D | | | | | | | |
| D:I-2 | 1958 | - | - | - | - | - | - |
| D:II-2 | 1981 | GLILD | - | Splenomegaly | Normal | - | - |
| Case E | | | | | | | |
| E:II-1 | 1992 | Bronchiectasis | Enlarged as a child; biopsies unremarkable | Splenomegaly (previous ITP) | Normal | - | - |
| Case F | | | | | | | |
| F:II-1 | 1946 | Bronchiectasis, fibrosis | - | - | - | - | - |
| Case G | | | | | | | |
| G:II-1 | 1980 | Bronchiectasis, fibrosis | - | - | - | - | - |
| Case H | | | | | | | |
| H:II-1 | 1973 | - | - | Splenomegaly | - | Chronic diarrhea with villous atrophy | CMV retinitis |
| Case I | | | | | | | |
| I:II-1 | 1991 | Bronchiectasis | Enlarged (mediastinal) | Splenomegaly | Mild elevation transaminases, gamma-GT | - | - |
| Case J | | | | | | | |
| J:III-2 | 1969 | Bronchiectasis (2005)  Asthma | - | - | Normal | Intermittent diarrhoea and abdominal pain | - |
| Case K | | | | | | | |
| K:II-1 | 1952 | Bronchiectasis (2009) | Previous peripheral T cell lymphoma: CD3+CD8+ (2015) | Splenomegaly (previous AIHA) | - | - | - |
| Case L | | | | | | | |
| L:II-1 | 1969 | Chronic left lower lobe collapse, COPD (smoker), no bronchiectasis | - | Splenectomy (AIHA) | Normal | - | - |
| Case M | | | | | | | |
| M:II-1 | 1985 | Bronchiectasis.  No interstitial disease.  Nodule top left lung | - | 'Mild' splenomegaly on CT only | Normal | Autoimmune enteropathy (2011) | - |
| Case N | | | | | | | |
| N:II-1 | 1959 | NAD | Generalised Lymphadenopathy | Large splenomegaly | Normal | - | - |
| Case O | | | | | | | |
| O:II-1 | 1978 | NAD | - | - | Normal | - | - |
| Case P | | | | | | | |
| P:II-1 | 1961 | GLILD  bronchiectasis | - | Splenomegaly (2012), normalized over time | Nodular regenerative hyperplasia of the liver. | Chronic diarrhea: upon biopsies absence of plasmacells (Norovirus-positive). | - |

**Abbreviations:**

AIHA: Autoimmune haemolytic anaemia; GLILD: Granulomatous-lymphocytic inflammatory lung disease; ITP: immune thrombocytopenia; NAD: No active disease

**Table E3. Immunological findings in individuals carrying *NFKB1* variants**

| **Case ID** | **Year of birth** | **Absolute lymphocyte count** | **Abs # CD3+ T cells** | **Abs # CD3/CD4+ T cells** | **Abs # CD3/CD8+ T cells** | **Abs # CD16/56+ NK cells** | **Abs # CD19+ B cells** | **IgA level (g/L)** | **IgG level (g/L) - prior to Ig subst.** | **IgM level (g/L)** | **ANA,**  **other autoAbs** |
| --- | --- | --- | --- | --- | --- | --- | --- | --- | --- | --- | --- |
| Case A | | | | | | | | | | | |
| A:II-1 | 1961 | 1.427 | 0.978 (68.5%) | 0.624 (43.7%) | 0.332 (23.3%) | 0.228 (16.0%) | 0.215 (15.0%) | 0.13 | 1.5 | 0.44 | ANA neg |
| A:II-4 | 1963 | 3.288 | 1.974 (60.0%) | 1.305 (39.7%) | 0.630 (19.2%) | 0.981 (29.9%) | 0.323 (9.8%) | 0.03 | 12.1  (post IVIG) | 0.44 | ANA neg |
| A:III-2 | 1989 | 1.630 | 1.400 (85.9%) | 0.403 (24.7%) | 0.941 (57.8%) | 0.153 (9.4%) | 0.074 (4.5%) | <0.04 | 6.9 | 0.17 | ANA neg |
| A:III-3 | 1995 | 2.527 | 2.269 (89.8%) | 0.735 (29.1%) | 1.401 (55.5%) | 0.252 (10.0%) | 0 (<0.50%) | <0.04 | 6.2 | <0.03 | ANA neg |
| Case B | | | | | | | | | | | |
| B:I-1 | 1939 | 1.189 | 0.820 (69,0%) | 0.240 (20.0%) | 0.540 (44.0%) | 0.060 (5.0%) | 0.290 (25.0%) | <0.1 | <0.05 | 0.26 | Coomb’s pos |
| B:II-1 | 1968 | 1.936 | 1.551 (80.1%) | 1.257 (64.9%) | 0.284 (14.7%) | 0.077 (4.0%) | 0.291 (15.01%) | 0.1 | 1.7 | 0.36 | Anti-TPO pos |
| Case C | | | | | | | | | | | |
| C:I-2 | 1938 | 1.741 | 1.316 (75.6%) | 0.243 (14.0%) | 0.943 (54.2%) | 0.375 (21.5%) | 0.047 (2.7%) | <0.1 | 2.0 | 0.6 | - |
| C:II-3 | 1972 | 0.950 | 0.870 (91.6%) | 0.450 (47.4%) | 0.410 (43.2%) | 0.060 (6.3%) | 0.020 (2.1%) | <0.1 | 1.5 | 0.20 | IDDM1 |
| C:II-5 | 1972 | 1.150 | 0.980 (85.2%) | 0.650 (56.5%) | 0.331 (28.8%) | 0.100 (8.7%) | 0.042 (3.7%) | <0.1 | 1.4 | 0.20 | - |
| C:III-1 | 1999 | 2.959 | 2.027 (68.5%) | 1.038 (35.1%) | 0.867 (29.3%) | 0.306 (10.3%) | 0.597 (20.2%) | 0.8 | 10.4 | 0.7 | - |
| C:III-3 | 2001 | 3.458 | 1.919 (55.5%) | 1.156 (33.4%) | 0.591 (17.1%) | 0.687 (19.9%) | 0.749 (21.7%) | 0.6 | 6.4  (low IgG2) | 0.2 | - |
| C:III-4 | 2004 | 4.054 | 2.615 (64.5%) | 1.565 (38.6%) | 0.821 (20.3%) | 0.514 (12.7%) | 0.883 (21.8%) | 1.3 | 5.7  (low IgG2 and IgG3) | 0.7 | - |
| Case D | | | | | | | | | | | |
| D:I-2 | 1958 | 2.5 | 1.373 (54.9%) | 0.761 (30.4%) | 0.553 (22.1%) | NA | 0.173 (6.9%) | 2.3 | 11.0 | 0.84 | - |
| D:II-2 | 1981 | 1.200 | 0.679 (55.6%) | 0.504 (42.0%) | 0.140 (11.7%) | NA | 0.027 (2.2%) | <0.3 | 4.9 | <0.1 | - |
| Case E | | | | | | | | | | | |
| E:II-1 | 1992 | 1.046 | 0.963 (92.1%) | 0.709 (67.8%) | 0.203 (19.4%) | NA | 0.022 (2.1%) | <0.1 | 3.9 | <0.1 | - |
| Case F | | | | | | | | | | | |
| F:II-1 | 1946 | 0.619 | 0.458 (74.0%) | 0.174 (28.1%) | 0.283 (45.7%) | NA | 0 | <0.1 | <2 | <0.1 | - |
| Case G | | | | | | | | | | | |
| G:II-1 | 1980 | 1.022 | 0.684 (66.9%) | 0.449 (43.9%) | 0.235 (23.0%) | NA | 0 | <0.1 | <3.9 | <0.1 | - |
| Case H | | | | | | | | | | | |
| H:II-1 | 1973 | 5.458 (splenectomy) | 4.857  (89%) | 2.129  (39%) | 2.620  (48%) | 0.262  (4.8%) | 0.132  (3%) | 0.02 | 0.1 | 0.03 | Coomb’s pos  ANA pos |
| Case I | | | | | | | | | | | |
| I:II-1 | 1991 | 1.4 | 1.102 (78%) | 0.717  (51%) | 0.349  (25%) | 0.166  (12%) | 0.122  (7%) | <0.06 | 0.3 | 0.09 | - |
| Case J | | | | | | | | | | | |
| J:III-2 | 1969 | 1.7 | 1.336  (78.6%) | 0.706  (41.5%) | 0.602  (35.4%) | 0.019  (1.1%) | 0.318  (18.7%) | <0.07 | <1.0 | 0.17 | - |
| Case K | | | | | | | | | | | |
| K:II-1 | 1952 | 0.7 | 0.639  (97%) | 0.206  (31%) | 0.388  (59%) | 0.005  (1%) | 0.015  (2%) | 0.05 | <0.1 | <0.1 | Coomb’s pos |
| Case L | | | | | | | | | | | |
| L:II-1 | 1969 | 1.4 | 1.129 (77%) | 0.687  (47%) | 0.405  (28%) | 0.249  (17%) | 0.080  (5%) | <0.05 | 10.3  (post SCIG) | <0.05 | Coomb’s pos |
| Case M | | | | | | | | | | | |
| M:II-1 | 1985 | 1.0 | 0.902  (90%) | 0.621  (62%) | 0.223  (22%) | 0.055  (5.5%) | 0.028  (2.8%) | <0.04 | Low pre Ig replacement | 0.65 | Coomb’s pos, ANA neg |
| Case N | | | | | | | | | | | |
| N:II-1 | 1959 | 1.012 | 0.82  (81%) | 0.54  (53%) | 0.26  (28%) | 0.15  (16%) | 0.02  (2%) | <0.07 | 2.3 | 0.18 | ANA neg |
| Case O | | | | | | | | | | | |
| O:II-1 | 1978 | 1.7 | 1.39  (82%) | 0.97  (57%) | 0.40  (24%) | 0.06  (3.5%) | 0.24  (14%) | 0.4 | 4.6 | 0.3 | ANA neg |
| Case P | | | | | | | | | | | |
| P:II-1 | 1961 | 0.79 | 0.52 (66%) | 0.33  (42%) | 0.18  (23%) | 0.11  (14%) | 0.15  (19%) | <0.1 | 1.9 | 0.1 | Coomb’s pos  ANA neg |

**Abbreviations:**

NA: Not Available
