# Supplementary material for: Loss-of-function nuclear factor κB subunit 1 (NFKB1) variants are the most common monogenic cause of common variable immunodeficiency in Europeans
Source: J Allergy Clin Immunol. 2018 Oct;142(4):1285–96. doi: 10.1016/j.jaci.2018.01.039 (PMC6148345; doi:10.1016/j.jaci.2018.01.039)
Supplement: Legends for Figures E1-E10 [file mmc3.doc]

**Supplemental Figure Legends**

**Figure E1. IGV plot of large *NFKB1* deletion**. IGV plot of patient I:II-1 showing the large *NFKB1* deletion identified by 50% reduction in the number of reads from the whole genome sequencing data mapping to that region.

**Figure E2. Numbers of CVID patients per gene in which we identified a likely pathogenic variant fully explaining the patient’s phenotype**. Assessment of all 390 CVID cases in our cohort identified 31 patients with a monogenic defect in 11 different genes. Variants in *NFKB1* contributed to more than half of all CVID patients with a monogenic diagnosis (16/31, 52%). *NKFB2* and *BTK* were the next most commonly implicated genes, with three explained cases each.

**Figure E3. Protein model of high-impact missense variants in and proximal to the ANK domain.** Residues observed with missense variants containing a high CADD score (≥20) are highlighted. Most of these are situated on the protein exterior (green) and appear equally in the primary immunodeficiency cohort and non-primary immunodeficiency cohorts (**Figure 2)**. A623G (#19) appears to be located more interior, although mutation of an Alanine to Glycine is a moderate substitution. Equally notable are residues of the Ankyrin repeats which previously have been probed as interaction sites upon NF-κB dimerization. While R614 (#7), K684 (#8), L517 (#13) and R687 (#16) could be considered part of these putative interaction sites, variants at these sites are found in non-primary immunodeficiency patients (**Figure 2**).

**Figure E4. Western blot analysis of all tested *NFKB1* variant carriers.** Western blot analysis targeting p50, IκBα and GAPDH of *NFKB1* variant carriers. Twelve patients with truncating variants (Arg284*, His513Glnfs*28, c.160-1G>A and Asp451*), one patient with gene deletion (del 103370996-103528207) and three patients with putative protein destabilizing missense variants (Ile281Met, Val98Asp and Ile87Ser) were tested. Relative fluorescence quantification of p50 and GAPDH by Odyssey Infrared Imaging system above and below each western blot. IκBα was not targeted in the Case D western blot.

**Figure E5. Serum IgM, IgG and IgA levels in serum of *NFKB1* LOF variant carrier**. Each dot represents an *NFKB1* LOF variant carrier and their age. In grey age-dependent reference values.

**Figure E6. Gating strategy for Figure 5B-E and additional B cell analyses**. **(A)** Representative flow cytometry plots of a healthy control, patient A:II-1 (clinically unaffected) and patient A:II-4 (clinically affected). Phenotype of CD19^+^CD20^+^ B lymphocytes. Numbers represent percentages in corresponding quadrants. **(B)** Percentages of CD27^−^IgD^+^ (naïve) or CD27^−^IgD^+^CD24^+^CD38^+^ (transitional) B cells. (*HD* healthy donor, *NFKB1*^+/-^ individual with *NFKB1* LOF variant.) Only individuals with sufficient B cells could be analyzed. P-values were determined by two-way ANOVA with Bonferroni post-hoc test (naïve B cells) or Student’s t-test (transitional B cells), *ns* not significant, **P≤0.01.

**Figure E7.** **Gating strategy Figure 6A and 6B and formation of CD38^+^ plasmablasts and IgA production.** **(A)** Representative flow cytometry plots of a healthy control and patient A:II-4 (clinically affected) after a 6 day culture of CFSE-labeled lymphocytes normalized for B cell number unstimulated, CpG/IL-2 (T cell independent activation) and anti-IgM/anti-CD40/IL-21 (T cell dependent activation). B cells gated on CD19^+^CD20^−/+^ and subsequently on CD27^++^ (left) and CFSE and CD38 (right). Numbers represent percentages in corresponding quadrants. **(B)** Plasmablast formation measured by proliferation and CD38 upregulation (CFSE^−^CD38^+^). **(C)** IgA production in the supernatant. (*HD* healthy donor; *CU* clinically unaffected or *CA* clinically affected individuals with LOF variant in *NFKB1*.) Only individuals with sufficient B cells could be analyzed. P-values were determined by two-way ANOVA with Bonferroni post-hoc test, *ns* not significant, **P≤0.01, ***P≤0.001.

**Figure E8. Additional lymphocyte numbers in individuals with *NFKB1* LOF variants.** Absolute numbers of total lymphocytes (CD45^+^), CD4^+^ and CD8^+^ T cells, NK cells (CD3^−^CD16^+^CD56^+^) and invariant natural killer T cells (CD3^+^Vα24^+^Vβ11^+^). Each dot represents a single individual and their age. In grey age-dependent normal values.

**Figure E9. Normal T cell differentiation in individuals with NF-κB1 deficiency.** **(A)** Representative flow cytometry dot plots of a healthy control and patient E:II-1, defining the differentiation of CD3^+^CD4^+^ and CD3^+^CD8^+^ T lymphocytes with CD27 and CD45RA. **(B, C)** Summary of subsets of **(B)** CD4^+^ T cells and **(C)** CD8^+^ T cells. (*HD* healthy donor, *NFKB1*^+/-^ individual with *NFKB1* LOF variant.) P-values were determined by two-way ANOVA with Bonferroni post-hoc test, *ns* not significant.

**Figure E10. T cells of individuals with *NFKB1* variants (*NFKB1*^+/-^) show normal proliferative capacity.** 6 day culture of CFSE-labeled lymphocytes unstimulated, anti-CD3/anti-CD28 (T cell receptor stimulation) or IL-15. Percentage of CD4^+^ or CD8^+^ T cell specific cell division as measured by CFSE dilution. (*HD* healthy donor, *NFKB1*^+/-^ individual with *NFKB1* LOF variant.) P-values were determined by two-way ANOVA with Bonferroni post-hoc test, *ns* not significant.
